# The plastid genome sequence of the invasive plant common Ragweed (*Ambrosia artemisiifolia*, Asteraceae)

**DOI:** 10.1080/23802359.2017.1390423

**Published:** 2017-10-18

**Authors:** Ali Amiryousefi, Jaakko Hyvönen, Péter Poczai

**Affiliations:** a Finnish Museum of Natural History (Botany), University of Helsinki, Helsinki, Finland;; b Department of Bioscience (Plant Biology), Viikki Plant Science Centre, University of Helsinki, Helsinki, Finland

**Keywords:** Chloroplast genome, *de novo* assembly, genome skimming, phylogenomics, plastid evolution

## Abstract

In the current study, we present the complete chloroplast genome sequence of *Ambrosia artemisiifolia*. The genome is 152,223 bp long and consist of 83 protein coding genes, 38 tRNAs, and four rRNAs duplicated in the inverted repeat. Detected large single-copy (LSC) and small single-copy (SSC) regions separated with two inverted repeat regions (IR) of length 25,098. The phylogenetic hypotheses obtained based on the analyses of 18 cp genomes places common ragweed within the tribe Heliantheae of the Asteraceae.

*Ambrosia artemisiifolia* L. (common ragweed) is one of the most prevalent invasive plants. The main concern regarding this plant is its large production of wind dispersed pollen that causes allergy for 15–60% of the European population (Taramarcaz et al. 2005). One single plant can produce six billion pollen grains and thousands of seeds during its lifecycle (Kazinczi et al. 2008). Besides its major health impact, it is causing serious economical problems for crop yield in agriculture. Yield losses alone were estimated to be €130 million per year for certain countries (Kőmíves et al. 2006). Ragweed is a plant of concern especially now in the era of warming climate because tests have shown that higher levels of carbon dioxide will greatly increase pollen production, and thus raising the number of people suffering of allergic reactions (Virág et al. 2016).

We extracted DNA according to Shi et al. (2012) from 20 g fresh ragweed leaves collected in Serbia (43.276149, 21.902976; voucher P.Poczai 0012867). Paired-end libraries of 2 × 150 bp were prepared with Illumina TruSeq DNA Sample prep kit and sequencing was carried out on an Illumina MiSeq platform. Raw reads were filtered with Trimmomatic (Bolger et al. 2014), and *de novo* assembly of the plastid genome was carried out with the Geneious R10 assembler platform (Kearse et al. 2012). We annotated the genome using Geneious and in-house scripts. Here, we report the complete chloroplast sequence of *Ambrosia artemisiifolia* to provide resources for taxonomic studies and invasive weed biology.

The complete chloroplast genome of *Ambrosia artemisiifolia* (GenBank accession MG019037) has a total length of 152,223 bp which is divided by two IR regions of 25,098 bp. This genome comprises of 125 genes and has 38% overall GC content. The genes are classified into 38 tRNA, 4 rRNA and 83 coding-protein genes.

Using the RAxMLv8.0 (Stamatakis 2014) the best scoring ML tree with 10,000 bootstrap replicates was calculated under GTR-GAMMA after running jModelTest2 (Darriba et al. 2012) including 16 representative species of the Heliantheae tribe (Asteraceae) and two outgroup species of Apiales (Figure 1). We also made phylogenetic analysis using parsimony as an optimality criterion and obtained similar topology. The same matrix was analyzed also with parsimony as an optimality criterion using WinClada (Nixon 2002) and TNT (Goloboff et al. 2008). Prior to the analysis we used the WinClada command ‘Mop uninformative characters’ to exclude parsimony uninformative characters. This resulted in a matrix with 30,216 characters and due to its small size, we were able to perform analyses using implicit enumeration of the TNT that ensures finding optimal tree(s). Phylogenetic hypothesis obtained by our study supported previous topologies (Panero and Funk 2008).

**Figure 1.**
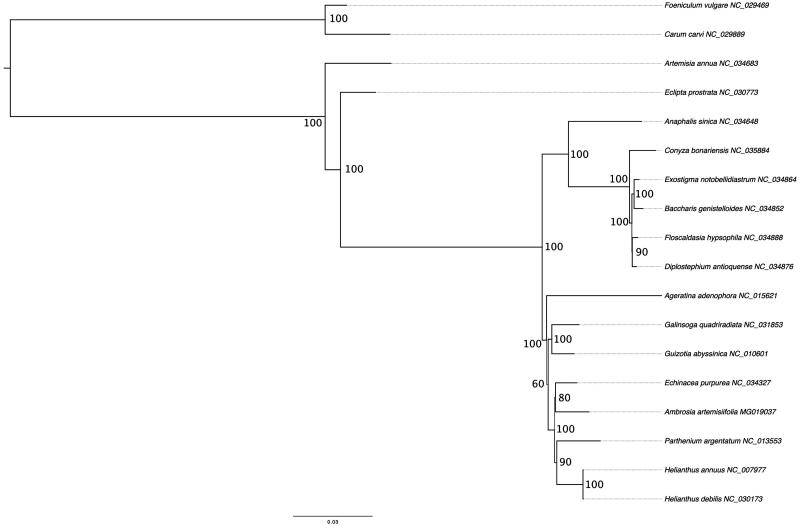
The ML tree of 18 selected chloroplast genome sequences and *Ambrosia artemisiifolia*. The values on the node show the bootstraps of 10,000 replicates and scale is substitution per site.

We expect this sequence to clarify the taxonomic status of the *Ambrosia* genus within the separate Ambrosiinae subtribe, and provide additional genomic resources for invasive plant genomics.
